# The Lesser of Two Weevils: Molecular-Genetics of Pest Palm Weevil Populations Confirm *Rhynchophorus vulneratus* (Panzer 1798) as a Valid Species Distinct from *R. ferrugineus* (Olivier 1790), and Reveal the Global Extent of Both

**DOI:** 10.1371/journal.pone.0078379

**Published:** 2013-10-15

**Authors:** Paul F. Rugman-Jones, Christina D. Hoddle, Mark S. Hoddle, Richard Stouthamer

**Affiliations:** 1 Department of Entomology, University of California Riverside, Riverside, California, United States of America; 2 Center for Invasive Species Research, University of California Riverside, Riverside, California, United States of America; University of Arkansas, United States of America

## Abstract

The red palm weevil (RPW) is a major pest of palms. It is native to southeast Asia and Melanesia, but in recent decades has vastly expanded its range as the result of multiple accidental anthropogenic introductions into the Middle East, Mediterranean Basin, Caribbean, and U.S.A. Currently regarded as a single species, *Rhynchophorus ferrugineus* (Olivier), RPW displays remarkable color variation across its range, and consequently has a taxonomic history littered with new species descriptions and synonymization. We compared DNA sequences of the mitochondrial cytochrome oxidase subunit I (COI) gene from RPW populations throughout the native and invaded ranges, to investigate the specific status and invasion history of this serious economic pest, and to identify possible common routes of entry. Analyses of COI haplotype data provide conclusive support, corroborated by sequences of additional nuclear gene regions, for the existence of at least two predominantly allopatric species. The true *R. ferrugineus* is native only to the northern and western parts of continental southeast Asia, Sri Lanka and the Philippines, and is responsible for almost all invasive populations worldwide. In contrast, the second species, which is currently synonymized under *R. ferrugineus* and should be resurrected under the name *R. vulneratus* (Panzer), has a more southern distribution across Indonesia, and is responsible for only one invasive population; that in California, U.S.A. The distribution of COI haplotypes is used to discuss the possible existence of further cryptic species, sources and routes of entry of different invasive populations, and the implications of our findings for current control methods.

## Introduction

The red palm weevil (RPW), *Rhynchophorus ferrugineus* (Olivier 1790) (Coleoptera: Curculionidae), is widely considered to be the most damaging insect pest of palms (Aracaceae) in the world [1–4]. The native range of RPW is thought to be restricted to Southeast Asia and Melanesia, stretching: through the countries bordering the Bay of Bengal from Sri-Lanka to the Malayan peninsula and Singapore; through Thailand, Cambodia and Vietnam; across the South China Sea to Taiwan and the Philippines; and down through the Sunda Islands (Java, Sumatra and Borneo) [1]. However, over the last 30 years, as the result of accidental introductions via the movement of live infested palms (e.g., coconut and date palms), RPW has undergone a huge range expansion, successfully invading the Middle East, the Mediterranean Basin, the Caribbean, China and Japan [3]. In August 2010, RPW was for the first time recovered from a dying Canary Island date palm (*Phoenix canariensis*) in the city of Laguna Beach, Orange County, California, USA [5,6].

**Figure 1:**
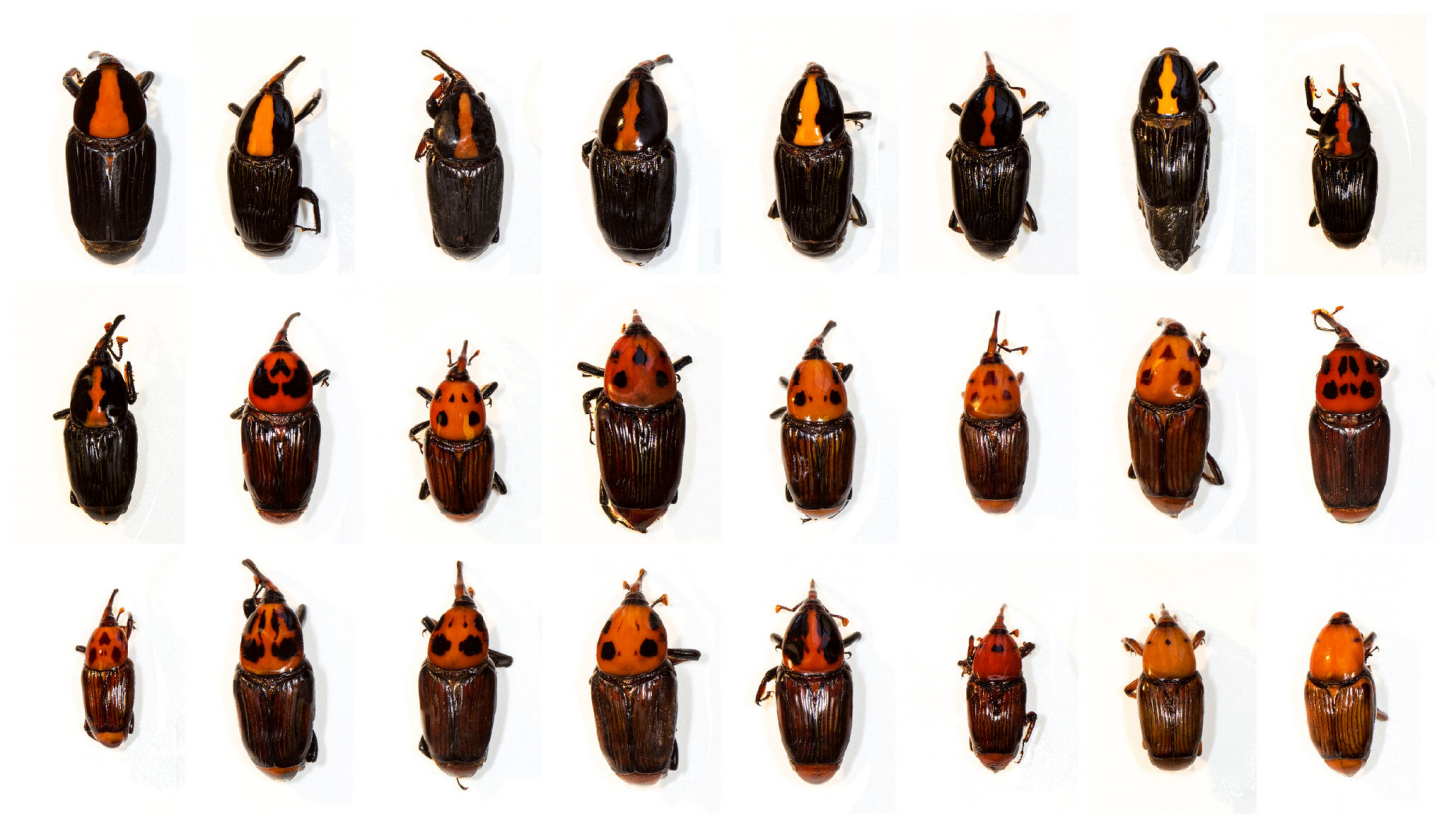
The extremes of color polymorphism in adult specimens currently classified as red palm weevil, *Rhynchophorus* *ferrugineus*. Molecular genetic data presented herein confirms that these specimens in fact represent at least two species, *R. ferrugineus* and *R. vulneratus*.

Adult RPW are large (30-40 mm in length) but display a high degree of color polymorphism. This color polymorphism has challenged taxonomists and other researchers for over two centuries. Currently, two color-morphs of RPW are recognized as a single species, *R. ferrugineus*: a “*ferrugineus*” (or orange with black markings) color morph and a “*vulneratus*” (or black with a red stripe) color morph (Figure 1) [7]. Almost the entirety of the invaded range of RPW has been colonized by the “*ferrugineus*” form. The only exception is California, where all specimens that have been recovered are the “*vulneratus*” form. The names given to these forms (“*ferrugineus*” and “*vulneratus*”) reveal something of the complicated taxonomic history of RPW. Wattanpongsiri [1] provides a detailed description of the early taxonomic history and the following provides a very brief chronologically ordered summary of his account (see Wattanpongsiri [1] for references). *Curculio ferrugineus*, was initially described by Olivier in 1790, from specimens collected in India, and transferred to *Rhynchophorus* by Herbst (1795) when he erected the genus. A second species, *Cordyle sexmaculatus*, was also described from India by Thunberg in 1797. *Rhynchophorus vulneratus* was first described, as *Curculio vulneratus*, by Panzer in 1798, although the locality attached to the specimen he described (South America) is almost certainly a mistake. Three years later, Fabricius (1801) described palm weevils from Sumatra as *Calandra schach*. This species was later transferred to *Rhynchophorus* (Schoenherr 1826), and in 1838, Gyllenhal also transferred *vulneratus* to *Rhynchophorus*, and recognized all three (*ferrugineus*, *schach* and *vulneratus*) as separate species. In 1845, without clear explanation, Boheman placed *ferrugineus* 1790 and *vulneratus* 1798 in synonymy with *schach* 1801 and also described *Rhynchophorus pascha* from the Philippines. Chevrolat complicated matters further in 1882, retaining *ferrugineus* and *pascha* in the genus *Rhynchophorus*, but dropping *vulneratus* and *schach*, and newly describing *Rhynchophorus signaticollis* and *Rhynchophorus indostanus* from Sri Lanka (formerly Ceylon) and the Indian state of Assam respectively. In 1885, Schaufuss added another species, *Rhynchophorus glabrirostris* from the Sunda Islands. In 1908, Heyne and Taschenberg, again without clear explanation, placed *schach* and *vulneratus* in synonymy with *ferrugineus*, but in the 1920s, during intensive studies of the biology of RPW, Corbett (1924, 1932) and Corbett and Ponniah (1923, 1924), again made reference to two species, consistently referred to as *schach* and *ferrugineus*. In 1936, Csiki placed *Cordyle sexmaculatus* in synonymy with *R. ferrugineus* and in 1956, Vestal again recognized the two species, *ferrugineus* and *schach*. 

This historical confusion eventually led Wattanapongsiri to conduct a thorough taxonomic revision of the genus *Rhynchophorus* (and the related genus *Dynamis*), which was published in 1966 [1]. After looking at thousands of adult specimens, from many locations throughout Southeast Asia and Melanesia, held in museum collections around the world, he recognized the synonymy of many of the existing names (e.g., identifying *schach* as a synonym of *vulneratus*), concluding that *ferrugineus* and *vulneratus* were the only valid species. Wattanapongsiri [1] produced detailed keys and descriptions and proposed that *R. ferrugineus* and *R. vulneratus* could be distinguished from each other primarily by the shape of the pronotum, and to a lesser extent by the color of the body. However, the specific status of *R. ferrugineus* and *R. vulneratus* was again brought into question in the 1990s, when researchers attempting to isolate aggregation pheromones, reported finding no differences in the chemical composition of pheromones produced by these species, or in the response of each species to the pheromones [8,9]. At the site of their studies, west Java, these researchers also reported the common occurrence of weevils that had color markings intermediate between those of *R. ferrugineus* and *R. vulneratus* (approximately 10% of captured specimens) [8]. In their review of RPW, Murphy and Briscoe [10] again questioned the validity of the two species, calling for the incorporation of DNA-based fingerprinting techniques to determine the relationship. Hallett et al. [7] returned to west Java and in 2004, published a more detailed investigation of the status of *R. ferrugineus* and *R. vulneratus* which included: re-examination of Wattanapongsiri’s morphological characters [1]; molecular genetic analyses based on both DNA sequences and RAPD (Random Amplification of Polymorphic DNA) banding patterns; and, a cross-breeding study. In their comparisons, Hallet et al. found that *R. ferrugineus* and *R. vulneratus* were alike in their morphological characters, mitochondrial DNA sequences (identical across a 201bp section of the cytochrome oxidase gene), and RAPD banding patterns [7]. Fertile F1 hybrids were also produced from a single heterospecific cross, and in light of these findings and their previous pheromonal work, they synonymized the two species, the name *R. ferrugineus* taking priority [7]. 

Debate over the specific status of *R. ferrugineus* has continued and over the last decade, interest in genetic variation, particularly among invasive populations of RPW, has increased, driven largely by the devastating effect of the pest on the date palm industry in the Middle East and Maghreb region of north Africa, and on ornamental Canary Island palms in the south east Mediterranean Basin. Early studies utilized RAPD methods to characterize genetic relationships among: different color-morphs drawn from a single collection site in Egypt [11] and Saudi Arabia (KSA) [12]; individuals from several locations within the United Arab Emirates (UAE), without reference to color [13]; and, individuals from Egypt, KSA and an Indonesian population [14]. Collectively, these studies suggested the existence of up to three genetic groups that were loosely correlated with color and/or collection location. In addition, KSA populations appeared to be more similar to those from Indonesia than those from Egypt. However, all of these studies were based on very small sample sizes, and taken from small geographic ranges, making it difficult to make any meaningful inference about phylogeography, genetic relationships, and the possible existence of cryptic species. 

In 2011, El-Mergawy et al. [15] made a more credible attempt to assess genetic variation in RPW, again utilizing RAPD markers to examine genetic variation among 27 populations, spread across 13 countries in the Mediterranean, Middle East, Pakistan and Japan. Although population sample sizes were again small (approximately 2 specimens per population), consideration of their data on a larger geographic scale revealed three phylogeographic groups: one containing all Egyptian and Mediterranean populations; another containing populations from the Middle East and Pakistan; and, a third made up of the Japanese population [15]. For the first time, these findings clearly suggested that populations of RPW in countries of the Mediterranean and the Middle East were founded by different invasion events. A second study, utilizing DNA sequences of the mitochondrial cytochrome *b* (Cytb) gene, but again with very small sample sizes, corroborated this finding, recovering two closely related haplotypes present in Middle Eastern and Pakistani populations, and a third more distantly related haplotype that was the only haplotype present in Mediterranean, Egyptian and surprisingly KSA populations, which had grouped with the rest of the Middle East using RAPDs [16]. The latter haplotype differed from the other two by approximately 3.5%, but the same study also found no variation in sequences of the ribosomal ITS2 gene, across the entire sample, suggesting that variation in Cytb was probably intraspecific rather than interspecific [17].

A third study, also published in 2011, considered variation in the DNA sequence of another mitochondrial gene, Cytochrome *c* oxidase sub-unit 1 (COI), and for the first time considered a reasonable number of samples; 310 specimens from 52 geographic localities across 14 countries in the invaded range [18]. The study resulted in the recovery of eight COI haplotypes which again clustered into distinct genetic groups according to geographic origin. Perhaps the most surprising finding was that only one haplotype, which was also the most genetically distant, was detected in the areas sampled in the Mediterranean Basin, Egypt and KSA. This mirrored the pattern shown by Cytb [16], but was this time represented by a much larger sample (a total of 198 specimens). Of the remaining haplotypes, one was exclusive to Japan and the remaining six were scattered throughout the Middle-East and Pakistan, but again with a high degree of population structure. Indeed, the presence of more than one haplotype was only detected in UAE and Oman [18]. One paradoxical finding was that Syrian populations of RPW (included for the first time) clustered with those in the Middle-East rather than their neighboring Mediterranean populations. These findings again suggested that the invasion of the Middle-East and Mediterranean (and Japan) by RPW followed different routes. The presence of only a single haplotype throughout the Mediterranean basin also suggested that the establishment of RPW in this region was likely the result of a single, and perhaps small, introduction, which subsequently seeded successive introductions from one country to another (including KSA). A less parsimonious alternative is the establishment of multiple introductions from a single, native source population. In addition, levels of COI sequence divergence between the Mediterranean haplotype and those found in the Middle-East and Japan (2.2 - 3.2%) were in the range that has been used to validate species boundaries in other taxa [19,20], again calling into question the specific status of *R. ferrugineus*.

In several cases, interpretation of existing studies that have attempted to examine molecular genetic variation in RPW, is hampered by their small sample sizes (see above). However, and more importantly, in every one of the published studies, the geographic range from which the samples were taken was also restricted. The molecular (and cross-breeding) data that contributed to Hallett et al.’s synonymization [7] was based on a small number of specimens collected from a small area, west Java, which lies within the vastly greater native range of RPW described by Wattanapongsiri [1]. In contrast, the majority of the Egyptian and Middle-Eastern studies [11–13,15,16,18] have focused only on RPW specimens and populations collected from the invaded range of the pest, where only the orange form is found. Indeed, only one study has made genetic comparison between a native and invasive population, but the study gives no details relating to exact sample locations and sizes, and contains several methodological shortcomings [14]. Here, for the first time, we conduct a rigorous examination of genetic variation in RPW populations from across the entire native and invaded range of this pest insect. We compare sequences of a 528bp section of the COI gene, revealing large amounts of variation across the native range, the existence of two very distinct genetic lineages, and evidence of strong phylogeographic structure across both the native and invaded ranges. With the addition of further sequence data from several nuclear genes, we provide conclusive evidence for the reinstatement of *R. vulneratus* as a valid species, highlight the possibility of additional cryptic species identifiable at the molecular level, and provide insight into the origins of the different invasive populations.

## Materials and Methods

### Ethics statement

Only one permit was required to complete the collections included in this study. Said permit was required for Singapore, where we had no official cooperators and collected only via traps placed on the balcony of a hotel room. Permit number NP/RP12-071 was issued by Jeremy Woon, Singapore National Parks Board. All other collections were made either with the direct permission of cooperating land owners (e.g., oil and coconut plantation owners/managers in Indonesia and Malaysia), or with the aid of university scientists and Ministry of Agriculture officials who had arranged access to their own study sites, or the private properties of cooperating landowners (e.g., Vietnam, Cambodia, Malaysia, Philippines, Pakistan, Saudi Arabia). In addition, a lot of material was sent to us by university researchers, government cooperators, and farm owners (e.g., Red Palm Weevil managers and scientists in India, Sri Lanka, Thailand, Egypt, Israel, France, Portugal, Spain, Italy and Cyprus). To the best of our knowledge none of the collections included herein were from National Parks or otherwise protected wilderness areas. Furthermore, these weevils are most certainly not an endangered species.

### Specimen collection

Collection sites throughout the native and invaded range varied greatly and included date plantations (e.g., KSA and Pakistan), forests (e.g., sago palms in Java), oil palm plantations (e.g., Papua New Guinea), coconut plantations (e.g., Philippines, Vietnam, and Malaysia), coconuts in oil palm plantations (e.g., Sumatra), and even the balconies of hotel rooms as high as the 18th floor (e.g., Cambodia and Singapore) (Table 1). As a result, no standard sampling strategy could be adopted. Instead, live RPW specimens were collected via one of three general methods, dictated largely by the surrounding habitat (Table 1). In the majority of cases, baited pheromone traps were deployed and adult specimens were collected daily. Traps incorporated a commercially available *R. ferrugineus* lure (P028-Ferrolure+700 mg; ChemTica International, Costa Rica) with an ethyl-acetate synergist (P080-Lure Weevil Magnet Pouch; ChemTica International), and were baited with either fermenting dates (in approximately 500 mL of water), or palm hearts extracted from trees (no water added) [21]. *Containers* used to house this material varied depending on the local availability of materials, but included 2 L plastic soda bottles, 5 L and 25 L plastic buckets, all of which had holes cut into them to allow RPW access to the lure and bait. In addition, or as an alternative, with the landowner's consent (and assistance), infested palms (sago, coconut, or date) were felled and "dissected" with chainsaws and machetes to reveal larvae, pupae, and adult RPW. Finally, in Thailand, RPW were also collected from commercial RPW production facilities where larvae are mass produced for food.

**Table 1:** Collection details.

**Country**	**Location**	**N**	**Collection Dates**	**COI haplotypes detected (see Figure 2)**
Aruba	Aruba	1	28-May-10	H20
Cambodia	Phnom Penh	2	27-Aug-12	H32
	Sihanoukville	4	27-Aug-12	H34
Curaçao	Curaçao	5	04 to 11-Mar-2011	El-Mergawy H8
Cyprus	Limassol	6	20-May-12	El-Mergawy H8, H33
Egypt	Paradise Park, 177 km south of Alexandria	2	Nov-11	El-Mergawy H8
France	Claret, Toulon	1	15-Nov-11	El-Mergawy H8
	Hyères, Almanarre	2	17-Nov-10	El-Mergawy H8
	Le Pradet	2	16-Nov-11	El-Mergawy H8
	Toulon	1	16-Nov-11	El-Mergawy H8
India	Duler, Mapusa, Goa	1	9-Jan-11	H16
	Loutolim, Goa	1	30-Dec-10	H9
	Majorda, Goa	1	30-Dec-10	H16
	Raia, Goa	1	2-Jan-11	H15
	Sao Jose de Areal (Nesai), Goa	1	1-Jan-11	H16
	Seraulim, Goa	1	29-Dec-10	H11
	Siolim, Goa	1	9-Jan-11	H14
Indonesia	Desa Tingan, Bali	8	2-Mar-12	Rv51, Rv52, Rv53, Rv54, Rv62
	Bunikasih, Java	21	24-Feb-11	Rv27, Rv28, Rv29, Rv30, Rv33, Rv36, Rv37, Rv39, Rv41, Rv59
	Cijangkar, Java	6	23-Feb-11	Rv26, Rv31, Rv32, Rv38, Rv42, Rv43
	Cikemang, Java	5	23-Feb-11	Rv32, Rv37, Rv40, Rv58
	Bah Lias Estate, nr. Pematangsiantar, Sumatra	18	24 to 29-Feb-2011	Rv12, Rv13, Rv14, Rv15, Rv17, Rv19, Rv21, Rv23, Rv24, Rv25
	Pancur Batu, Sumatra	10	25-Feb-12	Rv16, Rv18, Rv20, Rv22, Rv25
**Country**	**Location**	**N**	**Collection Dates**	**COI haplotypes detected (see Figure 2)**
Israel	Eylon	1	9-Jan-11	El-Mergawy H8
	Kerem Shalom	1	9-Feb-11	El-Mergawy H8
	Kfar Yuval	1	6-Feb-11	El-Mergawy H8
	Na'ama	2	9-Jan-11	H17
Italy	Campania (NA)	1	16-Sep-10	El-Mergawy H8
	Lazio (RM), Roma	3	09-Nov-2009, 27-Jan-2010, 26-Oct-2010	El-Mergawy H8
	Puglia (BA)	1	15-Sep-10	El-Mergawy H8
Malaysia	Rhu Tapai, Kuala Terengganu	27	3-Mar-11	El-Mergawy H8, H19, Rv9
	Sungai Burong	4	5-Jan-11	Rv1, Rv2, Rv4, Rv10
Pakistan	Bhawalpur, Punjab	4	25-Mar-11	El-Mergawy H5
	Muzaffargarh, Punjab	1	24-Mar-11	El-Mergawy H1
Papua New Guinea	Dami Research Station, West New Britain	2	2012	Rb7, Rb8
	Milne Bay Province	2	Aug-12	Rb5, Rb6
	Poliamba, New Ireland Province	11	11-May-12	Rb1, Rb2, Rb3, Rb4
Philippines	nr. San Pablo City, Luzon	22	23 to 26-Oct-2011	H23, 24, 25, 26, 27, 28, 29, 30, 31
Portugal	Ayamonte, *Andalucia*	1	5-Jan-11	El-Mergawy H8
	Tres Fontes, Algarve	1	7-Jan-11	El-Mergawy H8
Saudi Arabia	Al-Ahsa	6	29-Nov-2010 & 09-Mar-2011	H17, El-Mergawy H8
Singapore	Singapore	2	1-Sep-12	Rv6, Rv11
Spain	Benicassim	1		El-Mergawy H8
	Benimamet	1		El-Mergawy H8
	Luria	1		El-Mergawy H8
	Olliva	1		El-Mergawy H8
**Country**	**Location**	**N**	**Collection Dates**	**COI haplotypes detected (see Figure 2)**
Sri Lanka	Narammala, North Western Province	9	9-Aug-12	H10, H12, H13
Thailand	Bang Khan, Nakhon Si Thammarat Province	2	14-Oct-12	H22, Rv8
	Bang Kung, Huai Yot, Trang Province	8	8-Oct-12	H22, H43, Rv3, Rv6
	Chachoengsao Province	11	02 to 07-Aug-2012	El-Mergawy H8, H18, H19
	Chong, Trang Province	1	Mar-11	Rv7
	Kamphaeng Saen, Nakhon Pathom Province	1		H21
	Khao Kob, Huai Yot, Trang Province	1	1-Oct-12	H22
	La Mor, Trang Province	1	10-Oct-12	Rv5
	Na Khao Sia, Na Yong, Trang Province	2	4-Oct-12	H22
	Nakhon Pathom Province	2		El-Mergawy H8, H21
	Ta Pab, Trang Province	2	10-Oct-12	H22
	Tha Khae, Patthalung Province	4	4-Oct-12	El-Mergawy H8, H22
	Thung Tam Sao, Hat Yai, Songkhla Province	2	4-Oct-12	H22
Turkey	Antalya	1	16-May-11	El-Mergawy H8
	Didim	4		El-Mergawy H8
USA	Laguna Beach, California	8	21-Nov-10	Rv61
Vietnam	An Dihn Village, Mo Cày Nam District, Ben Tre Province	3	21-Aug-12	H36
	Hanoi	2	24-Aug-12	H37, H39
	Mo Cày Bac District, Ben Tre Province	2	21-Aug-12	H35
	Nga Tien Commune, Nga Son District, Thanh Hoa Province	7	24-Aug-12	H38, H40, H41, H42

 All RPW specimens were placed in labeled 50 mL, screw-capped conical centrifuge tubes (BD Biosciences, San Jose, CA), and euthanized by the addition of 95% ethanol. Each tube typically held 3-5 RPW specimens. The ethanol was replaced at least twice over the next 24-48h to ensure complete dehydration of tissues, thereby promoting adequate conditions for DNA preservation. Preserved samples were returned to our laboratory at the University of California Riverside (UCR), USA, and on receipt, RPW specimens were transferred to individual 20 mL clear PET sample vials (SKS Bottle & Packaging, Inc., NY), containing fresh 95% ethanol, and stored at -20 °C. 

 In addition to our field collected samples, a tissue sample (amounting to a single leg or small larval section) was obtained from each of eight RPW specimens held in the California State Collection of Arthropods, CDFA, Sacramento, that were collected in Laguna Beach, California, in 2010 (Table 1). While conducting our study, we were also contacted by a colleague in Papua New Guinea (PNG), offering us freshly collected material described as the *vulneratus* color-morph of RPW. On arrival at UCR, these specimens were instead found to match the description of *R. bilineatus*, common in PNG, but for confirmation and comparison, we included them in our study anyway. Finally, again to allow wider comparison, we also obtained samples of *R. cruentatus* and *R. palmarum* from Florida, U.S.A. and Tijuana, Mexico, respectively.

### DNA extraction and sequence generation

Whole genomic DNA was extracted from individual specimens using Chelex® 100 resin [22]. For adults and pupae, a small piece (2-5 mm^3^) of muscle tissue was dissected from a single tibia using flame-sterilized forceps, and allowed to air dry for 1 min. For larvae, a similar quantity of tissue was dissected from the head capsule and allowed to air dry. The tissue was transferred to a sterile 0.6 mL microcentrifuge tube and ground up in 6 µL proteinase-K (>600mAU/mL; Qiagen, Valencia, CA) using a micropestle. To this was added 120 µL of a 5% (w/v) suspension of Chelex® 100 resin (Bio-Rad Laboratories, Hercules, CA) and the reaction was incubated at 55 °C for 1 h followed by 10 min at 99 °C. The remainder of each extracted specimen (e.g., for adults, the entire body minus one tibia) is deposited in the Entomology Research Museum collection at UC Riverside (accession numbers: UCRC ENT 296876 - UCRC ENT 296902, UCRC ENT 378638 - UCRC ENT 378737, and UCRC ENT 378813 - UCRC ENT 378968).

 The polymerase chain reaction (PCR) was used to amplify a section of the mitochondrial gene (mtDNA) cytochrome oxidase c subunit 1 (COI) from each specimen. PCR was performed in 25 µL reactions containing 2 µL of DNA template (concentration not determined), 1X ThermoPol PCR Buffer (New England BioLabs, Ipswich, MA), an additional 1 mM MgCl_2_, 200 µM each dATP, dCTP, dGTP, 400 µM dUTP, 4% (v/v) BSA (NEB), 1 U Taq polymerase (NEB), and 0.2 µM of each PCR primer. Initial reactions utilized the primers Bron and Simon (El-Mergawy et al. 2011c) but it quickly became clear that no single primer pair worked with all our samples. Consequently, the majority of reactions used the primers C1-J-1718 and C1-N-2329 [23], which span a similar stretch of COI. If neither of these first two sets of primers worked, Bron and C1-N-2329 were combined in a third attempt. Reactions were performed in a Mastercycler^®^ ep gradient S thermocycler (Eppendorf North America Inc., New York, NY) using one profile for all 3 primer combinations: an initial denaturing step of 2 min at 94 °C; followed by five cycles of 30 s at 94 °C, 1 min 30 s at 45 °C, and 1 min at 72 °C; followed by a further 35 cycles of 30 s at 94 °C, 1 min 30 s at 51 °C, and 1 min at 72 °C; and, a final extension of 5 min at 72 °C. Amplification was confirmed by standard agarose gel electrophoresis and PCR products were cleaned using the Wizard® PCR Preps DNA purification system (Promega, Madison, WI) and direct-sequenced in both directions at the Institute for Integrative Genome Biology, UCR. PCR primers were trimmed and sequences were aligned using SEQUENCHER 4.9 (Gene Codes Corporation, Ann Arbor, MI). The absence of pseudogenes was confirmed by translating each sequence using the online EMBOSS Transeq tool [24].

 Based on the outcome of genealogical analyses of the COI sequences (see below and Results), we also examined sequence variation in two sections of nuclear ribosomal RNA (rRNA) for a subset of 46 specimens. The D2 domain of 28S rRNA (28S-D2) is generally highly conserved across related taxa, and small differences may be indicative of species boundaries [25]. Similarly, the faster evolving internal transcribed spacer 2 (ITS2) region is also typically conserved within a species, but it is likely to show greater variation between species [17]. Sections of 28S-D2 and ITS2 were amplified using the 28sF3633 and 28sR4076 primers and protocol detailed in Rugman-Jones et al. [25], and the ITS2-F and ITS2-R primers and protocol of Navajas et al. [26] respectively. Amplification products were cleaned and sequenced as above, and all mtDNA and rRNA sequences were deposited in GenBank^®^ (accessions KF311358-KF311740).

### Sequence analysis

**Figure 2:**
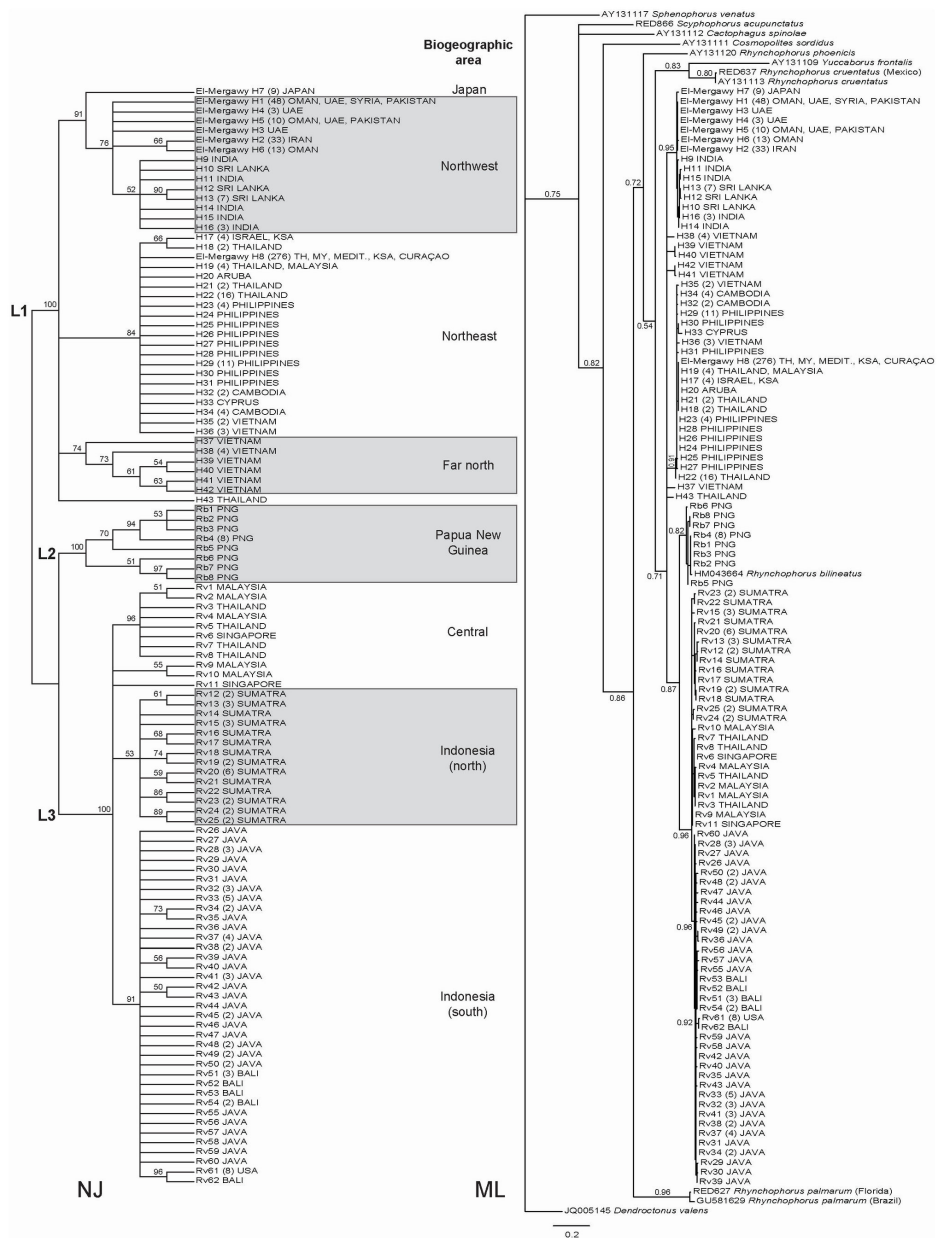
Strong phylogenetic and phylogeographic signal revealed by the genealogical relationships of COI haplotypes inferred using neighbour-joining (NJ) and maximum likelihood (ML) methods. Haplotypes fell into three lineages (L1-L3) and are numbered according to their position (top to bottom) within each lineage in the NJ tree (those in L1 follow on from the eight haplotypes of El-Mergawy et al. [2011c]). Bootstrap consensus NJ tree was computed using Kimura 2-parameter distances and 1000 replicates in MEGA 5.05. Branches with <50% support have been collapsed and in all other cases, bootstrap support is shown next to the branch. ML tree constructed in PhyML. Branch support was assessed using the approximate likelihood-ratio test (SH-Like) and branches with a probability below 0.5 have been collapsed. Support for major branches is shown next to the branch. Abbreviations (where used): TH=Thailand, MY=Malaysia, UAE=United Arab Emirates, KSA=Kingdom of Saudi Arabia, Medit.=Countries of the Mediterranean basin (including Egypt, but excluding Syria).

Sequences of the COI gene of 274 specimens matching the varied color descriptions of RPW, and 15 specimens matching the more precise color description of *R. bilineatus* were generated in this study. These were combined with, and trimmed to match, 310 existing RPW sequences [18] retrieved from GenBank (accession numbers GU581319-GU581628), resulting in a matrix of 599 sequences, each 528bp long. Sequences were collapsed into haplotypes, and the number and nature of polymorphic sites was characterized, using DnaSP v5.10.01 [27]. Sequence divergence between individual haplotypes was quantified as Kimura 2-parameter (K2P) distances calculated using MEGA 5.05 [28]. K2P distances were visualized by constructing a neighbor-joining (NJ) tree again using MEGA 5.05. Branch support for the NJ tree was assessed with 1000 bootstrap replicates. Genealogical relationships among haplotypes were also examined using maximum likelihood (ML) analyses, conducted with PhyML (v3.0 aLRT) [29], via the "Phylogeny.fr" platform [30]. Four further species of *Rhynchophorus*, five species from different genera in the same sub-family (Dryopthorhinae), and a “rooting” taxon from the sister sub-family (Scolytinae), were retrieved from GenBank (for accession numbers see Figure 2) and included in the ML analysis. The HKY85 substitution model was selected assuming an estimated proportion of invariant sites (of 0.505) and 4 gamma-distributed rate categories to account for rate heterogeneity across sites. The gamma shape parameter was estimated directly from the data (gamma = 0.568). Branch support was assessed using the approximate likelihood-ratio test (SH-Like) [31].

The NJ and ML analyses revealed strong evidence for the existence of at least three genetic lineages (designated: L1, haplotypes [El-Mergawy] H1-H43; L2, haplotypes Rb1-Rb8; and, L3, haplotypes Rv1-Rv62), and a clear signal in the geographic distribution of COI haplotypes within each lineage (see results, Figure 2). As a result, we divided the haplotypes into eight groups, corresponding to seven broad biogeographic areas within the described native range of RPW [1], and one in the invaded range. These were: “Northeast”, which included all haplotypes grouping with those from northern Thailand, Cambodia, Vietnam, and the Philippines; “Northwest”, which included all haplotypes grouping with those from Goa (India) and Sri Lanka; "Far north", which included haplotypes from Vietnam, north of the 17th parallel; “Central”, which included haplotypes from the Thai-Malay Peninsula and Singapore; "Indonesia (north)", which included haplotypes from Sumatra; “Indonesia (south)”, which included haplotypes from Java and Bali; “Papua New Guinea”; and finally, “Japan” (see Table 1 and Figure 2). Sequence divergence within and between these biogeographic areas was quantified as mean K2P distances using MEGA 5.05.

Ribosomal RNA sequences were aligned using MUSCLE 3.7 [32] via the "Phylogeny.fr" platform [30]. GenBank sequence matches were sought using standard nucleotide BLAST searches [33] and differences were simply quantified in terms of numbers of substitutions and insertions/deletions. 

### Morphometrics

**Figure 3:**
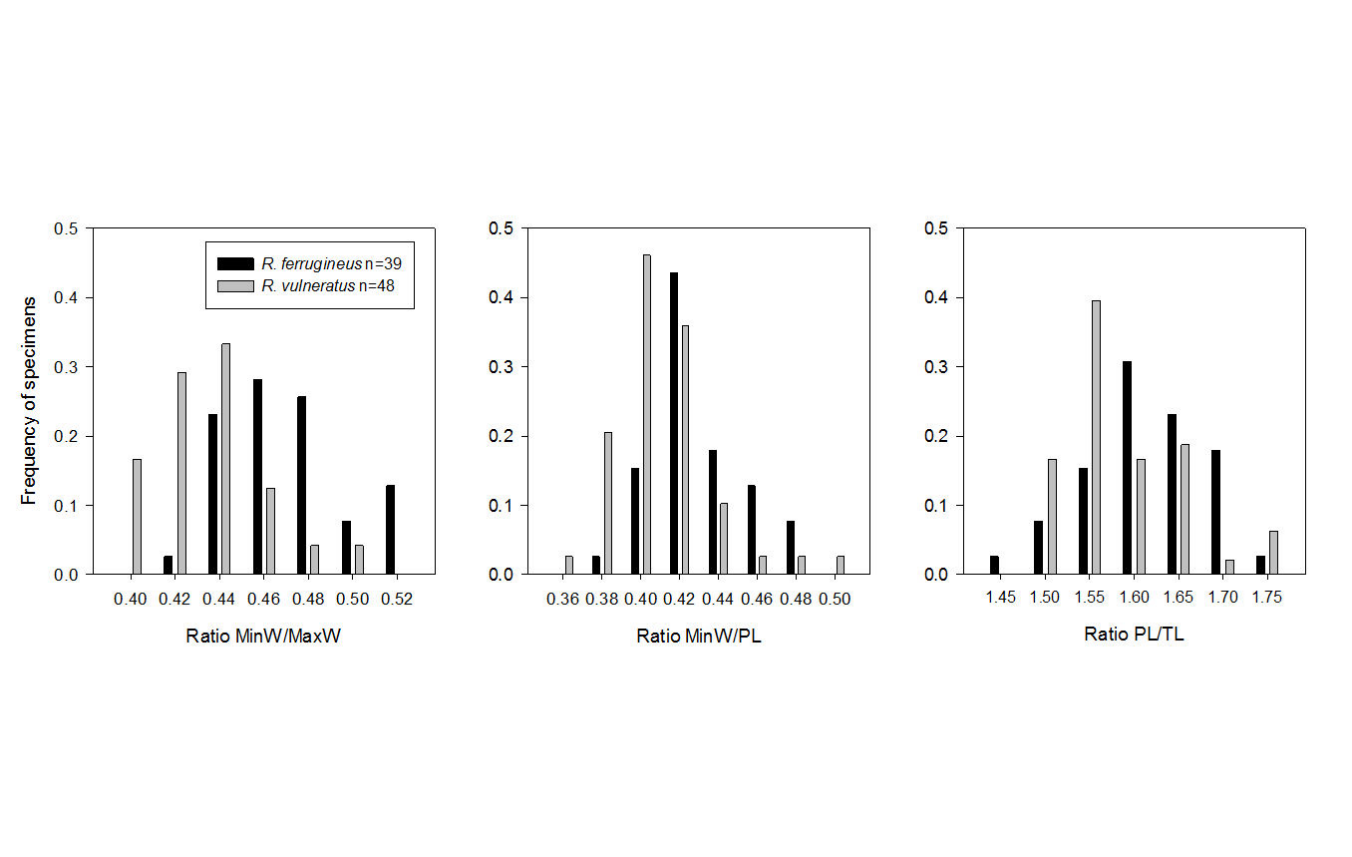
Overlapping distributions of three estimates of pronotal shape in red palm weevils from lineages L1 and L3.

Wattanpongsiri [1] suggested that *R. ferrugineus* and *R. vulneratus* could be distinguished by the shape of the pronotum. With the hindsight of our genetic analyses (see Results), the shape of the pronotum of 89 sequenced adult specimens across lineages L1 and L3 (Figure 2) was investigated. Weevils were mounted dorsal side up and photographed under a Leica Z16 APOAF microscope (Leica Microsystems, Wetzlar, Germany), using a JVC KY-F75U 3CCD digital camera (LVC Americas Corp., Wayne, New Jersey). Individual images of the pronotum were captured at several focal depths using ARCHIMED v.5.4.1 (Microvision Instruments, Évry, France), and subsequently stacked using CombineZP (http://hadleyweb.pwp.blueyonder.co.uk/CZP/News.htm) using the "Do Stack" algorithm. Measurements were taken from the stacked images using ARCHIMED. Following Hallett et al. [7], four measurements were taken from each specimen (maximum and minimum width, pronotal length, and the length of a transect from the midline at rear to the point at which the maximum width line met the antero-lateral margin (see Figure 3 [7]), and converted to three estimates of pronotal shape: ratio of minimum to maximum pronotal width (MinW/MaxW); ratio of minimum pronotal width to pronotal length (MinW/PL); and, ratio of pronotal length to transect length (PL/TL). Under these estimates, a specimen with a square pronotum will produce a MinW/MaxW ratio close to 1.0, while a lower value indicates that the pronotum is more vertically oval or circular; a vertically oval pronotum is indicated by a low MinW/PL, while a large value indicates a horizontally oval specimen; and, a high PL/TL ratio indicates an oval pronotum, while a lower ratio indicates a circular or square pronotum. Evidence of differences in pronotal shape between the two lineages was sought via ANOVA and Tukey's pairwise comparisons at the 0.05 level of significance, with lineage and sex as a single combined factor, using Minitab® 15.1.30.0 (Minitab Inc., State College, PA).

## Results

Among the COI sequences of 599 individual weevils (289 from the present study and 310 from El Mergawy et al. [18]), collected from locations across the native and invaded range of RPW, we found a total of 135 polymorphic sites, 11 non-synonymous substitutions, and 113 haplotypes (Figure 2). The NJ tree based on K2P distances grouped these haplotypes into three distinct lineages with 100% bootstrap support (designated L1 – L3, Figure 2). Haplotype codes (and corresponding museum vouchers) of individual specimens are annotated in the sequence information uploaded to GenBank (accession numbers KF311360-KF311648). ML analyses recovered the same three lineages, again with a high level of support (>0.7) (Figure 2). One lineage (L2 in Figure 2) was strictly of Papua New Guinean origin and formed by haplotypes (designated Rb1 – Rb8) from specimens matching the morphological description of *R. bilineatus* (see methods). Furthermore, this lineage grouped with an existing GenBank accession for *R. bilineatus* (HM043664) in the ML analysis (Figure 2) and therefore, we are confident this lineage represents *R. bilineatus*. 

The remaining two lineages (L1 and L3, Figure 2) contained the haplotypes of specimens matching the morphological descriptions of RPW. Divergence between these lineages was large (> 13%), and included consistent non-synonymous substitutions at positions 215 and 247. Position 215 was cytosine in all haplotypes making up L1, but always adenosine in L3. Similarly, position 247 was adenosine in all but one haplotype (detected in only a single specimen) in L1, but always guanine in L3. Divergence in the COI gene was also corroborated by consistent differences in more conserved regions of ribosomal RNA. Across 493bp of the 28S-D2 from 46 representative specimens, we found no variation within each of the COI lineages (or indeed between L2 and L3), but 2bp difference between L1 and L3 (positions 251 and 428; GenBank accessions KF311649-KF311694). Across approximately 549bp of the more variable ITS2 we again found no variation within L1, and a very small amount of variation within L3 (positions 122, 296 and 307). However, L3 differed from L1 with 11-14 substitutions and a single base deletion. *R. bilineatus* was distinct from both L1 and L3 with a 24bp deletion between positions 397 and 420 inclusive, and by several further substitutions (GenBank accessions KF311695-KF311740). Taken together, this is very strong evidence that the L1 and L3 represent different species.

The two RPW lineages (L1 and L3) also displayed strong geographic structure (Figure 2). L1 contained all 8 haplotypes previously identified by El Mergawy et al. [18], plus a further 35 haplotypes identified from our samples (designated H9 – H43). It had a characteristic northern and western distribution and comprised all specimens from native populations in India, Sri Lanka, Pakistan, Cambodia, Vietnam and the Philippines, along with invasive populations in the Mediterranean, Middle East, Japan and West Indies. In contrast, L3, containing 62 haplotypes (designated Rv1 – Rv62), had a more southeastern distribution, and contained all our specimens from native populations in Singapore, the Indonesian islands of Sumatra, Java, and Bali, and the invasive population in California. The only geographic overlap between the two lineages occurred in southern Thailand and northern Malaysia on the Thai-Malay Peninsula.

Within each of the COI lineages, strong phylogeographic structure was also evident in the distribution of haplotypes (Figure 2), which we used to justify dividing the haplotypes into eight groups, corresponding to seven broad biogeographic areas within the described native range of RPW [1], and one in the invaded range (see Materials & Methods for group definitions). For example, all our specimens from India, Sri Lanka, and Pakistan shared haplotypes that clustered tightly, and were distinct from other L1 haplotypes (Figure 2). Similarly, in L3, all our specimens from Sumatra and Java harbored haplotypes that formed well supported clusters with no overlap between the two islands (Figure 2). Variation, expressed as mean K2P distances, between the haplotypes within each of the 8 groups was relatively low, with a maximum of 1.6% (Table 2). However, variation between groups was approximately 2- to 9-fold higher (Table 2).

**Table 2:** Mean Kimura 2-parameter distances within and between the COI haplotypes present in red palm weevil populations across 8 biogeographic areas.

	NE	NW	Far north	Japan	Central	Indonesia (north)	Indonesia (south)	PNG
NE	0.0107							
NW	0.0324	0.0099						
Far north	0.0250	0.0261	0.0037					
Japan	0.0366	0.0221	0.0269	0.0000				
Central	0.1358	0.1405	0.1271	0.1372	0.0143			
Indonesia (north)	0.1417	0.1459	0.1307	0.1417	0.0278	0.0128		
Indonesia (south)	0.1485	0.1509	0.1362	0.1485	0.0309	0.0269	0.0091	
PNG	0.1195	0.1319	0.1218	0.1311	0.0883	0.0885	0.0881	0.0163

### Morphometrics

Across specimens, there was a large amount of variation in all three estimates of pronotal shape (Figure 3). Within each lineage, there were no differences between males and females in the means of any of the three estimates and we also detected no significant difference between the lineages for the estimate PL/TL (Table 3.). For the estimate MinW/PL, females from lineage L1 were on average bigger than those from L3, but male size did not mirror this pattern. Only MinW/MaxW, revealed a consistent difference between lineages L1 and L3, with L1 on average having a more square shaped pronotum (i.e. larger MinW/MaxW) than L3, independent of sex (Table 3). However, there was still a large degree of overlap between the lineages in the distribution of our estimates of MinW/MaxW, rendering it useless for reliably diagnosing the lineages (Figure 3).

**Table 3:** Estimates of the shape of the pronotum of red palm weevils from lineages L1 and L3 (see Figure 2).

Lineage	Sex	*N*	MinW/MaxW ^†^ (mean ± SE)	MinW/PL ^‡^ (mean ± SE)	PL/TL ^§^ (mean ± SE)
L1	Female	21	0.468 ± 0.006 a	0.430 ± 0.005 a	1.609 ± 0.013 a
	Male	18	0.469 ± 0.006 a	0.426 ± 0.006 ab	1.618 ± 0.017 a
L3	Female	21	0.425 ± 0.005 b	0.408 ± 0.004 bc	1.572 ± 0.013 a
	Male	27	0.442 ± 0.005 b	0.411 ± 0.006 ac	1.596 ± 0.013 a

Values in the same column, followed by the same letter, are not significantly different (Tukey's pairwise comparisons, individual confidence level = 98.96%).

† ANOVA: F_3,83_=15.71, p<0.001

‡ ANOVA: F_3,83_=3.87, p=0.012

§ ANOVA: F_3,83_=1.94, p=0.129

## Discussion

In recent decades, RPW has undergone a huge range expansion, and now poses a serious threat to commercial date groves, ornamental landscape plantings and native stands of palm trees worldwide. Fundamental steps in any attempt to address this "threat" include a reliable understanding of the taxonomy of the pest, and identification of sources and routes of entry of invasive populations. In the taxonomic literature, RPW is currently considered to be a single species, *Rhynchophorus ferrugineus* [7]. The present study combined DNA sequence data from new material collected in its native range with existing sequences deposited in an open-access database (GenBank), to test this species hypothesis, and to examine genetic variation among RPW populations across the native and invaded ranges.

### Synonymy and taxonomy

Sequences of the mitochondrial COI gene, across almost 600 RPW specimens, revealed high levels of genetic variation and a total of 113 haplotypes. In turn, these haplotypes constituted three major, highly divergent (>10%) lineages (Figure 2). The same mitochondrial lineages were corroborated by differences in the sequences of two separate conserved regions of nuclear ribosomal RNA, 28S-D2 and ITS2, providing strong evidence for the existence of at least three different species. One of the lineages (L2, Figure 2) was only detected in specimens from Papua New Guinea and as expected (see methods), represents *Rhynchophorus bilineatus*. In our phylogenetic analyses, *R. bilineatus* fell between the remaining two lineages, and was consistently placed as a sister clade to lineage L3, and a more distant relative of lineage L1 (Figure 2).

**Figure 4:**
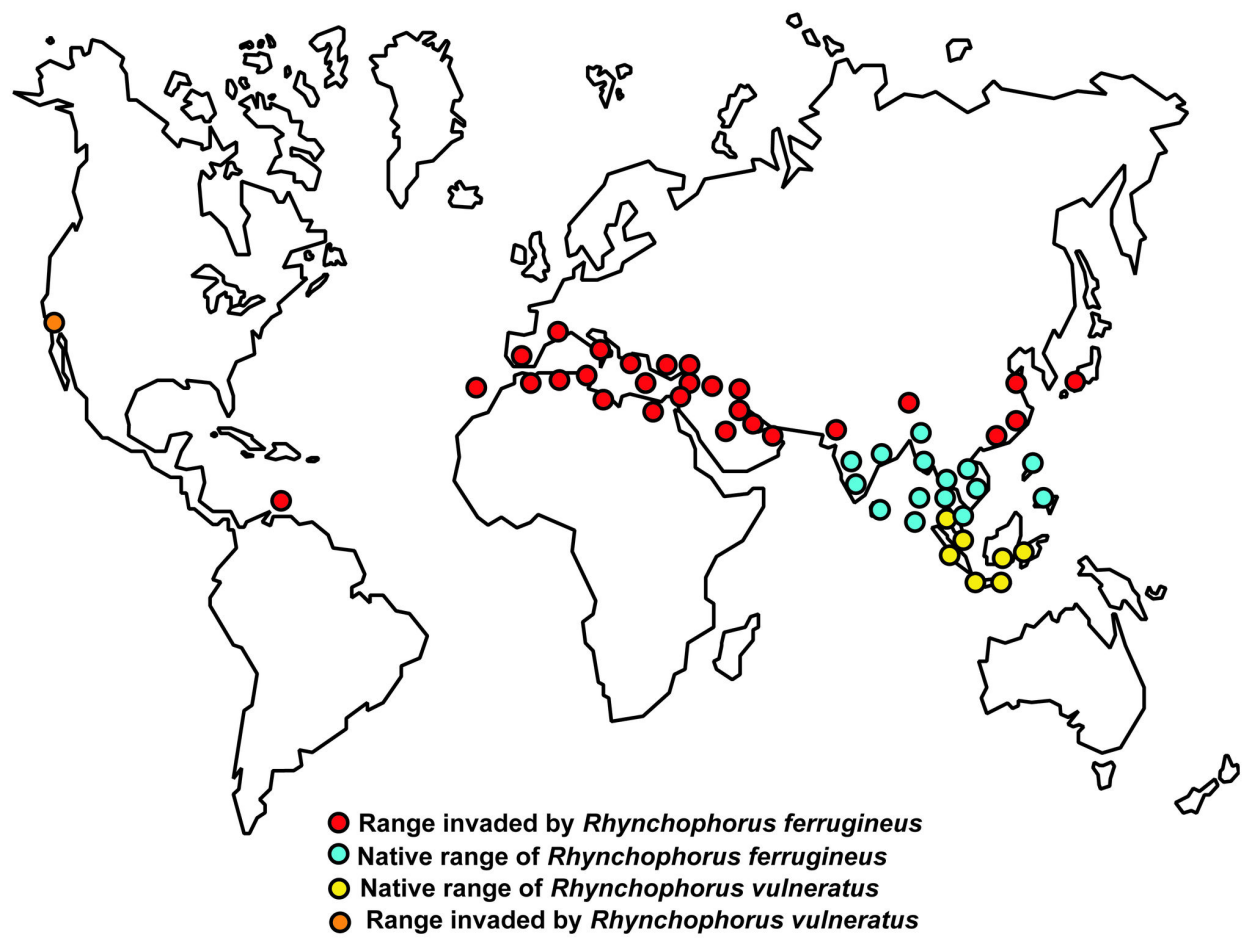
Global distribution of *R. ferrugineus* and *R. vulneratus.*

There was also very clear and correlative geographic division in the distribution of COI haplotypes between the L1 and L3 lineages (Figure 4). Within the native range, L1 haplotypes were characterized by their northern and western distribution, in India, Sri Lanka, Cambodia, Vietnam and the Philippines. In contrast, L3 haplotypes had a more southeastern distribution, residing in specimens from Singapore, and the Indonesian islands of Sumatra, Java, and Bali. These distributions are very close to those described by Wattanapongsiri [1] for *R. ferrugineus* and *R. vulneratus* respectively. In our study, the only detected geographic overlap between the two lineages occurred on the Thai-Malay Peninsula, around the border between Thailand and Malaysia (Table 1; Figure 4). Again, according to Wattanapongsiri [1], this is more or less exactly where the ranges of *R. ferrugineus* and *R. vulneratus* meet. Therefore, although morphological support remains elusive (see below), in light of the phylogeographic evidence, we propose that the name *Rhynchophorus vulneratus* (Panzer) be reinstated and applied to palm weevils from Singapore, Sumatra, Java and Bali. In so doing, we also propose that palm weevils to the north and east of the Thai-Malay Peninsula (i.e. lineage L1) are the "true" *Rhynchophorus ferrugineus* (Olivier) (but see below). Interestingly, the zone of overlap between the species (or lineages) is also close to the proposed site of two ancient seaways that bisected the Thai-Malay Peninsula, for durations in excess of 1 million years, during the Miocene (24-23 Mya) and Pliocene (5.5-4.5 Mya) eras; an area known as the Isthmus of Kra [34]. These ancient seaways may have acted as barriers to dispersal [34,35], restricting gene flow and contributing to divergence between *R. ferrugineus* and *R. vulneratus*. Wattanapongsiri [1] also suggests that the two species co-occur in the Philippines. We did not find any evidence of *R. vulneratus* in the Philippines, but we were only able to sample a single location on the island of Luzon, the northernmost region of the archipelago. Thus, it remains possible that *R. vulneratus* is found in southern parts of the Philippines (e.g., the island of Mindanao).

Across its native range there was also a relatively large amount of genetic variation across COI haplotypes within the *R. ferrugineus* (L1) lineage, dividing it into 3 sub-groups which differed from each other by 2.5 - 3.2% (Figure 2; Table 2). Again, there was a strong geographic component to this grouping. Although rRNA sequences were homogenous across all sampled individuals from this lineage, levels of divergence of around 3% in the COI sequence are often cited as indicative of species boundaries [19,20]. Thus it is possible that one or more of these subgroups represents a distinct cryptic species rather than a population of a single widespread species. Given the complicated taxonomic history of RPW, there is no shortage of candidates [1]. Further genetic work, involving a greater number of loci (see 36), and/or cross-mating studies would likely go a long way to revealing the existence of any cryptic species. Similar genetic structure was also evident in the distribution of *R. vulneratus* (L3) haplotypes across the Indonesian islands of Sumatra, Java, and Bali, with each of the 50 haplotypes detected in this region, being restricted to a single island, and haplotypes from Sumatra, and those from Java and Bali combined, forming separate clades (Figure 2). Average sequence divergence between these clades was around 2.7%, and again the possible existence of cryptic species warrants further investigation. 

**Figure 5:**
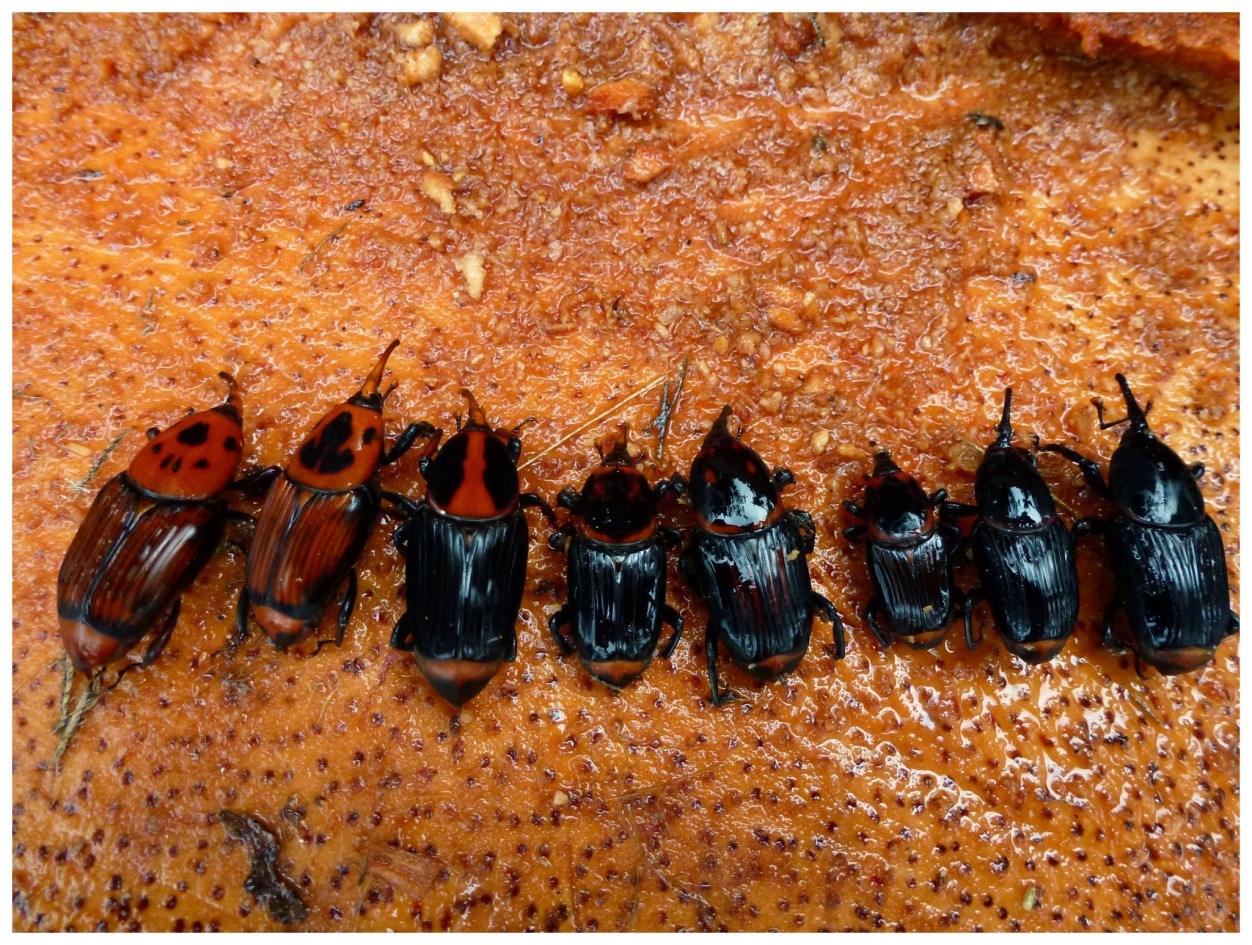
Color polymorphism in *R. ferrugineus* specimens captured in a single night in pheromone traps deployed in a commercial coconut plantation in Oct. 2011, Laguna Province, Luzon, the Philippines.

The distribution of the different RPW color-morphs was not so clear cut. Like almost all of those occurring in the invaded range, the majority of native specimens tentatively identified genetically as *R. ferrugineus* (lineage L1, Figure 2) were the orange color-morph. The exceptions to this were collections from the northern Philippine island of Luzon, specimens from which displayed a huge range of color variation from orange through to almost entirely black (Figure 5). In comparison, the colors displayed by specimens identified genetically as *R. vulneratus* were more variable, exhibiting the full range of color variation (see Figure 1). This is almost certainly what confused Hallett et al. [7,8] and lead to the incorrect synonymization of *R. ferrugineus* and *R. vulneratus*. There is no overlap between our COI sequences and those of Hallett et al. [7], but it seems that while they were correct in identifying the different color-morphs on the Indonesian island of Java as a single species, that species was almost certainly not *R. ferrugineus*. Indeed, in collecting their molecular and cross-breeding data, it is highly unlikely that they even encountered *R. ferrugineus*, and instead worked only with a *R. vulneratus* population exhibiting high levels of color variation. For an organism with such an expansive native range, this highlights the perils of conducting research to identify species within such a small geographic area. Color variation is also likely to have lead Wattanapongsiri [1] astray (and contributed to the later confusion). The list of *R. ferrugineus* specimens he examined includes approximately 150 specimens from Java. Again, we found no genetic evidence of *R. ferrugineus* in Java. Furthermore, in his revision, Wattanpongsiri also listed another *Rhynchophorus* species described from a single Sumatran specimen, *R. lobatus*. While it appears he never actually viewed the specimen, he states "...the original description fits both *ferrugineus* and *vulneratus*" [1; pg. 140].

Our estimates of pronotal shape were also found to be unreliable characters for diagnosing *R. ferrugineus* and *R. vulneratus*, as was proposed by Wattanapongsiri [1]. Hallett et al. [7] reported a similar finding. However, although this part of their study compared specimens from a wider area than just Java, it was still conducted without DNA-based knowledge of the identity of their specimens (i.e. species designations were based on color), and therefore, their comparisons are redundant.

### Invasion history

With the new knowledge and acceptance that what is currently known as RPW constitutes at least two species, it is clear that the huge range expansion in recent years has resulted almost entirely from the movement and establishment of just one of those species, *R. ferrugineus* (Figure 2) (though see above regarding the possibility of further cryptic speciation). The invasive populations are also genetically very poor, displaying a dramatic reduction in the number of COI haplotypes present. Such a genetic bottleneck is characteristic of populations following an invasion, and in species that show highly structured genetic variation across their native ranges, the haplotypes that do remain in an invasive population can point to the geographic origins of that invasive population. RPW became established in Middle Eastern countries by the mid 1980s [37]. Collectively, across the countries sampled by El-Mergawy [18] in this region (UAE, Oman, and Iran), only six COI haplotypes (referred to as El-Mergawy H1-H6 in our study; see Figure 2) were detected from 77 specimens. One of those haplotypes (El-Mergawy H1) was also the only one they detected in Syria and Pakistan. This already extensive sample lead us to not focus on adding further genetic data from these countries, but in our small sample from Pakistan, we also detected that same haplotype, plus another (El-Mergawy H5). In the Middle East, RPW is often referred to as the “Pakistani weevil” and the evidence suggests that Middle Eastern populations may have invaded from Pakistan. However, RPW is also considered to be invasive in Pakistan so the true origin of the invasion may lie elsewhere. Outside of Pakistan, we did not detect any of the six haplotypes in our sample of the native range. However, these haplotypes were most closely related to those detected (H9-H16) in our samples from the northwestern part of the native range, in Goa (India) and Sri Lanka (Figure 2). We were unable to access material from the majority of the northwestern part of the native range due to Indian claims of intellectual property rights over genetic resources, but it is likely that a more thorough sampling of this and neighboring countries would locate the haplotypes in question. Thus, we hypothesize that RPW reached the Middle East, by way of Pakistan, following a westward expansion route out of India. 

 Invasive populations of RPW in the Mediterranean Basin, Egypt and KSA are even more genetically depauperate. Indeed, a single COI haplotype (El-Megarwy H8) accounts for almost everything (Figure 2). RPW was first detected in the Sharqiya region of Egypt in 1992 [38] and is thought to have been introduced there in an importation of palm offshoots from UAE [37]. It is then believed to have been accidentally moved to southern Europe in shipments of live adult palms from Egypt to Spain, which, at the time, were imported without restriction to satisfy a very substantial demand for ornamental landscaping that existed in many southern European coastal cities [37]. The overwhelming predominance of the El-Megarwy H8 haplotype throughout the Mediterranean Basin and Egypt (in the combined sequence matrix generated by our own collections and those of the previous study [18]) provides strong support for the idea that the RPW population in Egypt indeed acted as a "bridgehead" for the invasion of the rest of the Mediterranean Basin. However, since neither the El-Mergawy H8 haplotype nor any closely related haplotypes have been detected in UAE, we can dismiss the earlier idea that Egypt was first invaded by RPW arriving from UAE. Instead, in our analyses the El-Mergawy H8 haplotype was detected within the native range, only in RPW populations from Thailand and Malaysia, indicating that these are much more likely to be the original source of RPW populations in the Mediterranean Basin. However, the El-Mergawy H8 haplotype was also closely related to a cluster of other haplotypes (H17-H36) from the neighboring countries of Cambodia and Vietnam to the north and east, and across the South China Sea in the Philippines. More intensive sampling in these areas may also uncover the El-Mergawy H8 haplotype. Furthermore, Egypt itself may have received RPW from an earlier "bridgehead" population in KSA, where RPW was first detected as early as 1986 [39], and the El-Mergawy H8 haplotype was also found. RPW larvae are very high in protein and interestingly, in parts of its native range the "sago worm" is commonly consumed as food. Indeed, in Thailand, RPW larvae are even commercially-reared for human consumption [40]. We sampled several mass-rearing facilities in Thailand and found that the El-Mergawy H8 haplotype was present. Following the establishment of relations in 1957, in a period until 1989, hundreds of thousands of Thais went to KSA to work [41]. Perhaps one of these immigrant workers introduced RPW to KSA to provide a ready, local supply of a traditional delicacy? 

 Support for a "bridgehead" population in KSA also comes from a second haplotype, H17, which was only found in KSA and Israel, where it didn't establish until 1999 [42]. Regardless, the distribution of COI haplotypes provides clear evidence that invasive RPW populations in the Middle East and the Mediterranean Basin initially originated from very different native populations (and may even constitute different cryptic species; see above). This is in line with the findings of previous studies that have revealed genetic differences between the two invasive populations [15,16,18], but unlike previous studies we have also narrowed the potential sources of each invasive population to specific regions within the native range.

 Secondary translocation of RPW from Egypt or the Mediterranean Basin is also the most parsimonious explanation for the presence of the El-Mergawy H8 haplotype on the island of Curaҫao in the Caribbean. However, a second haplotype (H20) was unique to the neighboring island of Aruba. At least three scenarios could account for the presence of this haplotype. First, the Caribbean was invaded just once and H20 is simply a very rare haplotype that was not detected in the combined dataset of sequences from Egypt, KSA or the countries of the Mediterranean Basin. Alternatively, since H20 differs from El-Mergawy H8 by only a single nucleotide substitution, the former haplotype may have arisen as the result of a mutation that occurred post-invasion. This would account for its absence elsewhere in our sample. Finally, perhaps the least likely scenario is that H20 is evidence of a second invasion event in the Caribbean, that coincidentally originated from a similar part of the native range. While the last scenario may be an unlikely explanation for a rare haplotype in the Caribbean, it may account for another rare haplotype in the invaded range. Haplotype H33 was found only in a single specimen (collected with others) from Limasol, Cyprus. Again, this haplotype clusters with El-Mergawy H8 (present in the other Cypriot specimens) but this time it differs from that haplotype at 11 positions (2.1%). This haplotype was not detected in our samples from the native range, but ML analyses indicate that it is most closely related to haplotypes from the Philippines (Figure 2). 

 Potential sources of the invasive RPW population in Japan, sampled by El-Mergawy et al. [15,18], remain elusive. Only a single haplotype (designated El-Mergawy H7 in the present study) was detected in Japan, and this haplotype suggests that the population is indeed *R. ferrugineus* (Figure 2). We identified its closest relatives as native haplotypes from India and Sri Lanka, and invasive haplotypes from the Middle East, but it was still somewhat distant from these haplotypes. Therefore, it seems likely that the Japanese population originated from an area in the north western part of the native range, not sampled in this study; perhaps in India, Bangladesh, or Myanmar.

 Finally, and in contrast to all other invasive populations, we can confirm that the palm weevils that invaded Laguna Beach, California, U.S.A., in 2010, were not *R. ferrugineus*, but *R. vulneratus*. Only a single COI haplotype (Rv61) was found among 8 specimens from California, and yet again, we were unable to detect an exact haplotype match in native populations of this species. However, Rv61 was very closely related to a group of haplotypes (Rv26-Rv62) from the Indonesian islands of Java and Bali, the latter island providing the closest match (a single nucleotide substitution between Rv61 and Rv62). The introduction of *R. vulneratus* seems unlikely to have resulted from permitted movement of live palm trees (in which the pest may have lain hidden as eggs, larvae, or pupae) since there have been are no palm plantings in the Laguna Beach area, and the USDA banned all palm imports at the beginning of 2010 (specifically to prevent RPW invasion). Palm smuggling is also an unlikely conduit since these weevils require large palms in which to live and breed. Thus, it is again possible that the weevils, in this case *R. vulneratus*, were deliberately introduced to the Laguna Beach area for food. Thankfully, at the time of publication, there had been no reported captures of RPW in Laguna Beach since January 2012 despite an extensive trapping effort from June-November of the same year. Furthermore, the recent removal and inspection (27th March 2013) of a Canary Island palm that had been previously inspected and treated for *R. vulneratus* infestation in 2011, found no evidence of weevil activity [43]. Thus, it appears that the small *R. vulneratus* population in Laguna Beach may have gone extinct.

### Aggregation pheromones, mass trapping and control

 In many invaded areas, mass trapping has been used to reduce RPW densities [2,3,10,39]. In this instance, aggregation pheromones are loaded into bucket traps along with bait (i.e., palm material or dates mixed with water to ferment), an ethyl acetate synergist, and granular insecticides (see 21). RPW adults, attracted by the pheromones, synergist, and bait, fly into the bucket trap and once inside, the pesticide kills the weevils before they can escape. Several companies synthesize commercial lures for RPW based on a male aggregation pheromone, ferrugineol, originally characterized by Hallet et al. [8]. While it is successful for trapping *R. ferrugineus*, in light of our findings, it seems most likely that ferrugineol was actually isolated from *R. vulneratus*. This raises the issue of why it works. It is possible that there are shared compounds in the aggregation pheromones of the two species that result in a similar end-product, which in the invaded range, since there is no alternative, *R. ferrugineus* is attracted to regardless. However, it is also possible that differing blends of these shared compounds, or the addition of further compounds, may produce a lure that is even more attractive to *R. ferrugineus*. Consequently, we strongly suggest that the aggregation pheromone for *R. ferrugineus* should be isolated, identified, synthesized, and compared to the commercially-available pheromone that was derived from *R. vulneratus* and is marketed for *R. ferrugineus* control.
